# Supplementary material for: Mucus Release and Airway Constriction by TMEM16A May Worsen Pathology in Inflammatory Lung Disease
Source: Int J Mol Sci. 2021 Jul 22;22(15):7852. doi: 10.3390/ijms22157852 (PMC8346050; doi:10.3390/ijms22157852)
Supplement: Supplementary file 1 [file ijms-22-07852-s001.zip › ijms-1281166-supplementary.pdf]

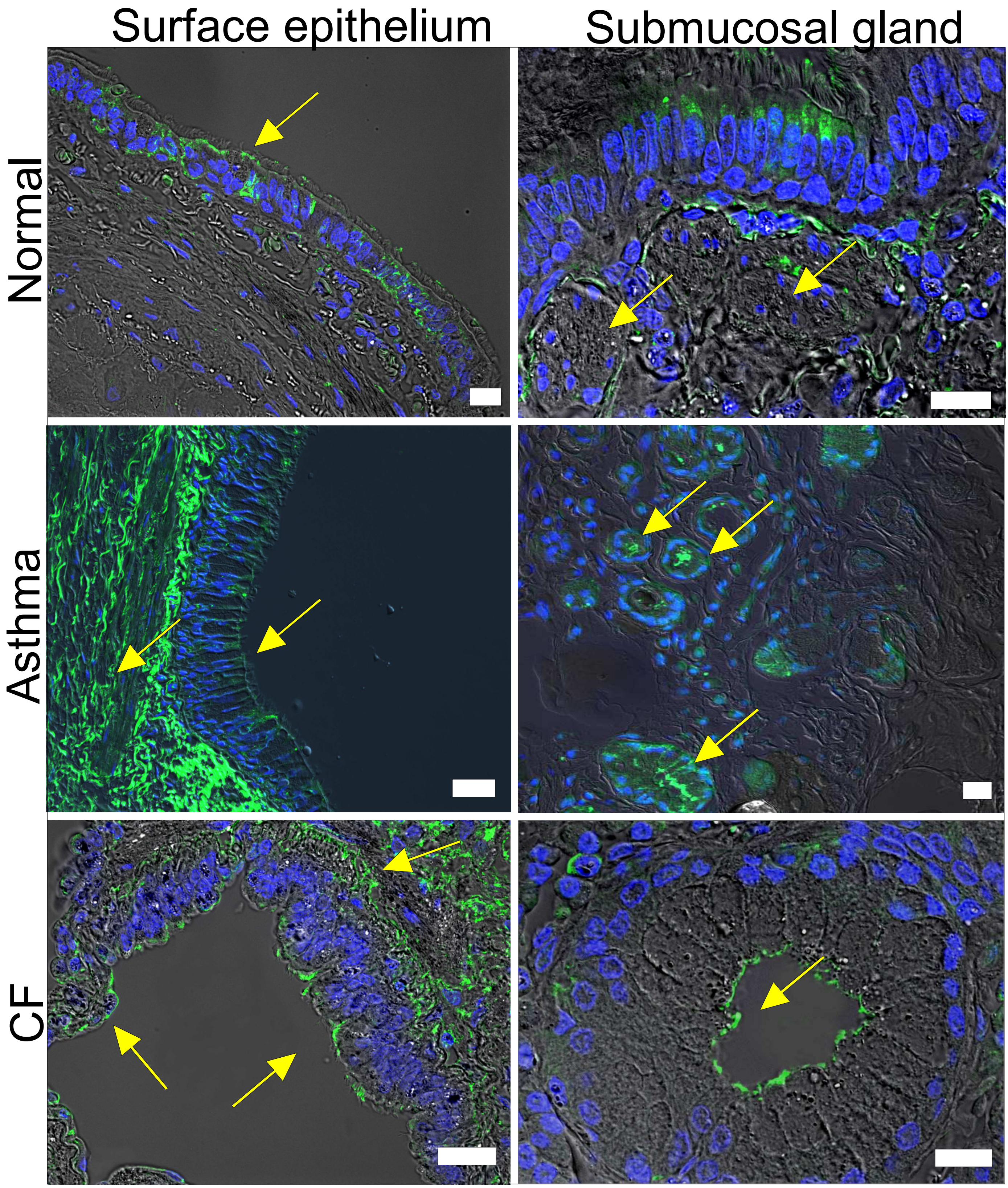

Figure E1. *TMEM16A is expressed in submucosal cells of human airways.* Immunofluorescence of TMEM16A in human airways. Little or no expression is found in surface epithelium, airway smooth muscle and submucosal glands of normal airways. Asthmatic airways show some expression in the surface epithelium, but clear expression in airway smooth muscle. Expression is also regularly detected in submucosal glands. CF airways show expression in surface epithelium and airway smooth muscle with higher levels of expression in submucosal glands. Bar = 20 µm. Yellow arrows indicate expression of TMEM16A (green fluorescence).

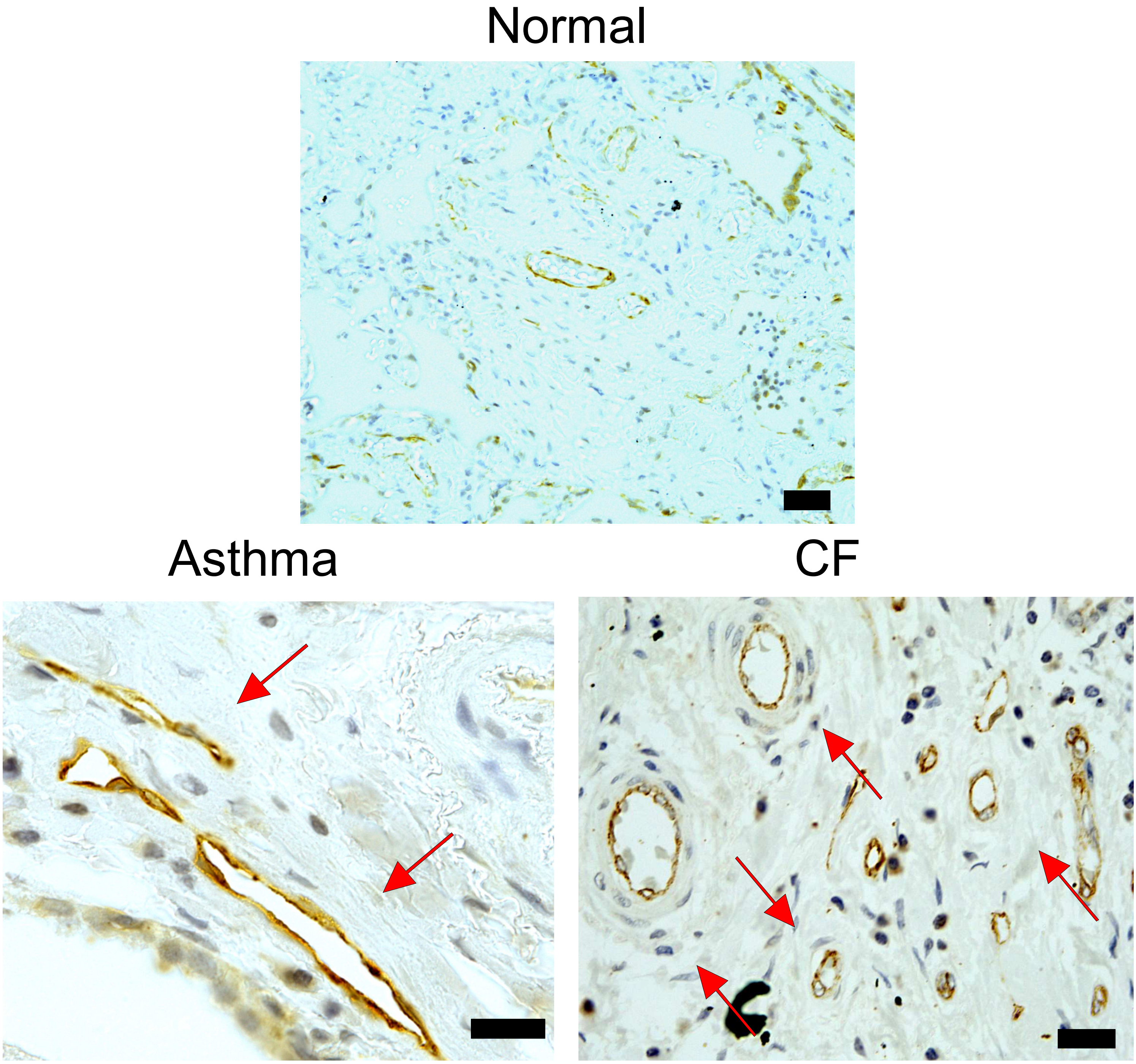

Figure E2. *TMEM16A is expressed in pulmonary blood vessels.* Using 3,3'-diaminobenzidine (DAB) staining, TMEM16A is found predominantly in the endothelium of pulmonary blood vessels. Pronounced staining (brown precipitation) is found in vessels of asthmatic lungs and lungs of people with CF. Bar = 20 µm. Red arrows indicate expression of TMEM16A (brown precipitation).

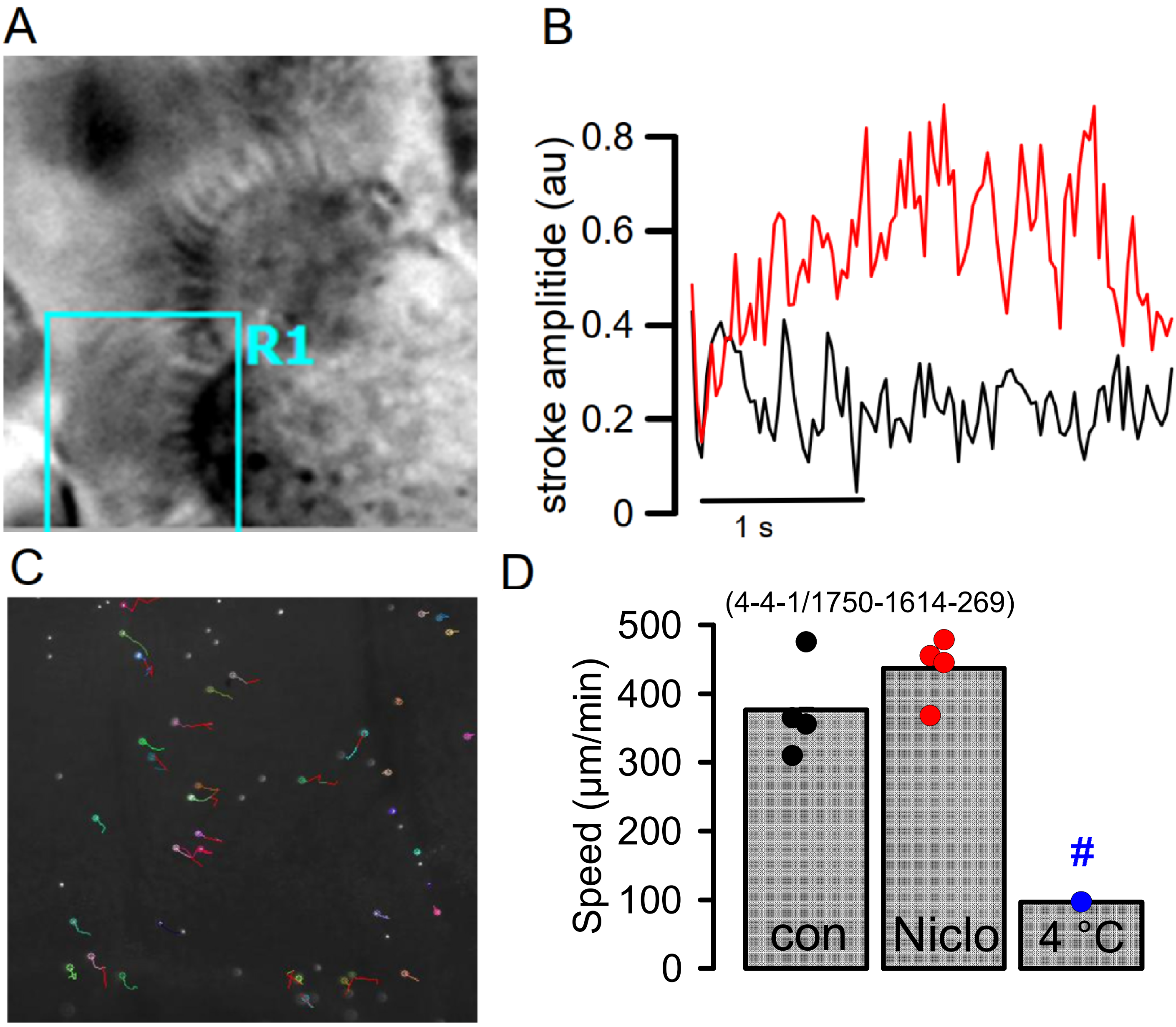

Figure E3. *Niclosamide does not inhibit mucociliary clearance.* A,B) Ciliary stroke amplitude and frequency measured and analyzed by video imaging of isolated mouse tracheas (Zeiss Axiovision, Munich). Acute application of 1 µM niclosamide does not affect stroke amplitude or frequency (n=3 mice/37-89 events). C,D) Particle tracking and trajectories assessed by video imaging of moving fluorescent beads on isolated tracheas. Experiments were performed in a humidified chamber. Niclosamide did not inhibit basal particle movement. In contrast, cooling of the tissue strongly reduced particle clearance. Mean ± SEM (n=4 mice/269-1750 particles tracked). [#]significant inhibition by low temperature ($p < 0.05$, ANOVA).

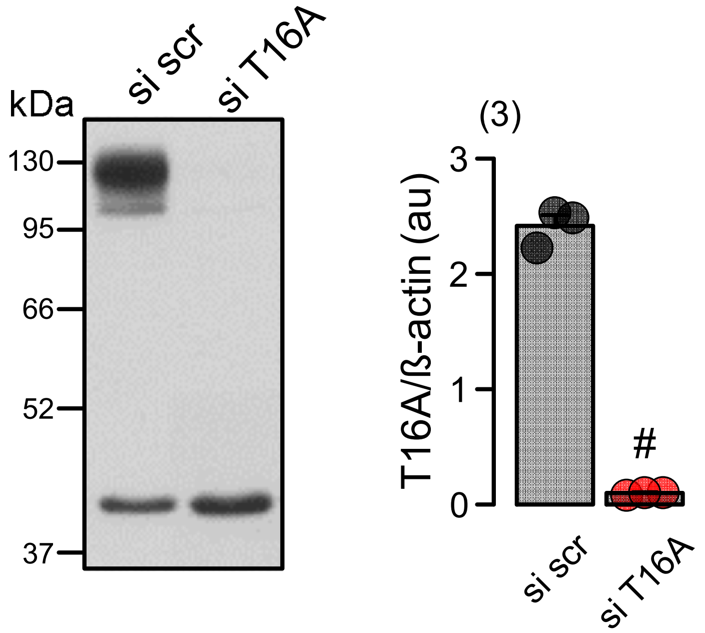

Figure E4. *siRNA-knockdown of TMEM16A in CFBE airway epithelial cells.* Western blot of TMEM16A expressed in CFBE airway epithelial cells treated with scrambled RNA or with TMEM16A-siRNA, which successfully knocked-down expression of TMEM16A (left panel). Blots were performed in 3 replicates (right panel). Mean±SEM (number of replicates). #significant difference ($p<0.05$; unpaired t-test).

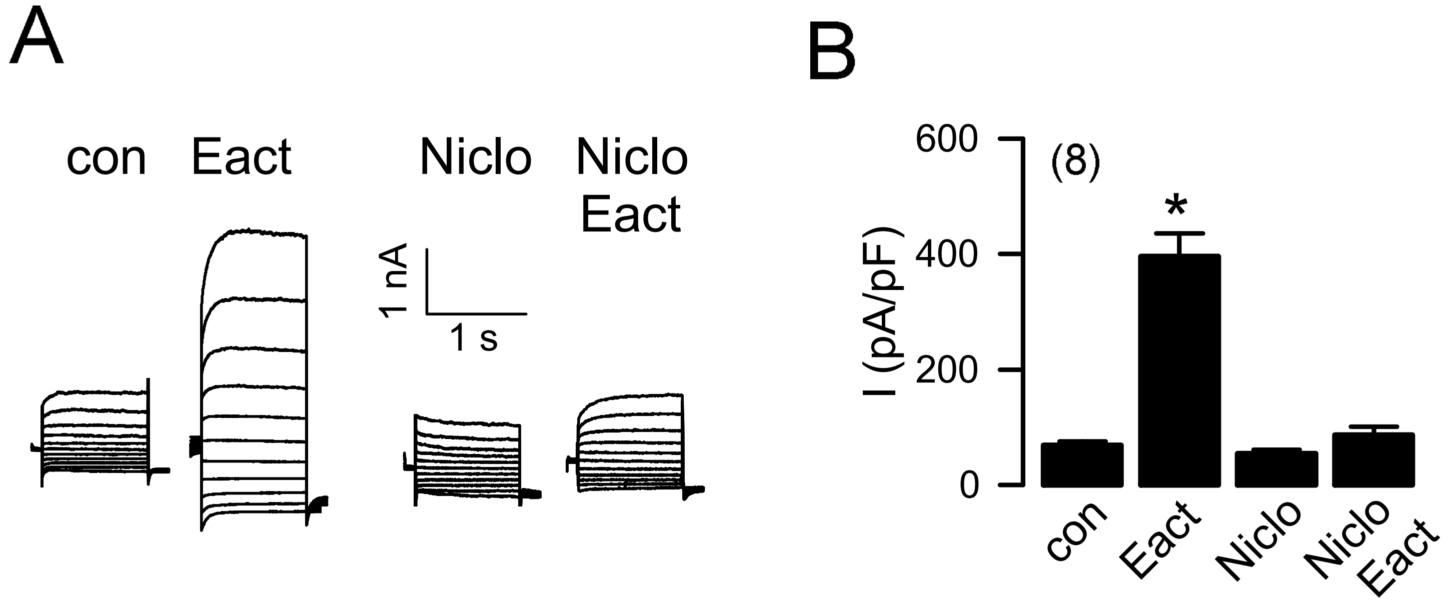

Figure E5. *Niclosamide inhibits activation of TMEM16A by Eact.* Expression of TMEM16A in HEK293 cells. TMEM16A whole cell currents were activated by the activator of TMEM16A, Eact (1 µM). Niclosamide (Niclo; 1 µM) completely inhibited activation of TMEM16A. Mean ±SEM (number of experiments). *indicates significant activation by Eact ($p<0.05$; paired t-test).
